# Is It Reliable to Take the Molecular Docking Top Scoring Position as the Best Solution without Considering Available Structural Data?

**DOI:** 10.3390/molecules23051038

**Published:** 2018-04-28

**Authors:** David Ramírez, Julio Caballero

**Affiliations:** 1 Instituto de Ciencias Biomédicas, Universidad Autónoma de Chile, 5 Poniente No. 1670, 3460000 Talca, Chile; david.ramirez@uautonoma.cl; 2 Centro de Bioinformática y Simulación Molecular (CBSM), Universidad de Talca. 1 Poniente No. 1141, 3460000 Talca, Chile

**Keywords:** rigid molecular docking, cross-docking, Glide, AutoDock, scoring energy, RMSD

## Abstract

Molecular docking is the most frequently used computational method for studying the interactions between organic molecules and biological macromolecules. In this context, docking allows predicting the preferred pose of a ligand inside a receptor binding site. However, the selection of the “best” solution is not a trivial task, despite the widely accepted selection criterion that the best pose corresponds to the best energy score. Here, several rigid-target docking methods were evaluated on the same dataset with respect to their ability to reproduce crystallographic binding orientations, to test if the best energy score is a reliable criterion for selecting the best solution. For this, two experiments were performed: (A) to reconstruct the ligand-receptor complex by performing docking of the ligand in its own crystal structure receptor (defined as self-docking), and (B) to reconstruct the ligand-receptor complex by performing docking of the ligand in a crystal structure receptor that contains other ligand (defined as cross-docking). Root-mean square deviation (RMSD) was used to evaluate how different the obtained docking orientation is from the corresponding co-crystallized pose of the same ligand molecule. We found that docking score function is capable of predicting crystallographic binding orientations, but the best ranked solution according to the docking energy is not always the pose that reproduces the experimental binding orientation. This happened when self-docking was achieved, but it was critical in cross-docking. Taking into account that docking is typically used with predictive purposes, during cross-docking experiments, our results indicate that the best energy score is not a reliable criterion to select the best solution in common docking applications. It is strongly recommended to choose the best docking solution according to the scoring function along with additional structural criteria described for analogue ligands to assure the selection of a correct docking solution.

## 1. Introduction

Molecular docking is a computational method that predicts the binding orientations of the ligands into a receptor-binding site; in this sense, it is a popular method in medicinal chemistry research [1]. It has become essential for developing different rational drug design protocols including structural-based virtual screening for identifying novel candidates and the understanding of the most important chemical elements that guide ligand-protein interactions in relevant biological targets [2]. The principal issues of docking are the accurate predictions of binding orientations and affinities [3]. For this, docking algorithms explore different ligand poses corresponding to different conformations and orientations within the receptor binding site, and they detect the “best docking solution” among these orientations by using a scoring function which evaluates binding energies [4]. There are many reports which have assessed and compared different docking protocols using different docking programs [5,6,7]. It is accepted in literature that docking methods can identify right solutions, since they reproduce X-ray crystallographic structures [8,9,10,11,12,13,14], however, the best solution is not always detected as the best ranked scored energy.

In general, docking predicts the preferred binding orientation of a ligand into a receptor [15,16,17]. The simpler case is when the docking of ligand is performed inside its own crystal structure, because the binding site have the optimal size to receive the ligand; we will refer to this process as a ‘self-docking’. On the other hand, docking is named ‘cross-docking’ when ligands are docked into receptor structure that is bound to a different ligand in the crystal structure. The last practice is more common, since docking is more relevant when it is used as a tool for investigating novel ligands. Previous reports comparing different docking protocols are commonly focused to self-docking tests; although it may highlight the work of Sutherland et al. [6], where cross-docking accuracy was assessed.

In a recent paper, we warned scientists about the use of docking predicted binding energies to compare the activities of pairs of molecules [15]. The present work is oriented to prevent about the selection of the best scoring energy as the best solution of a more common docking experiment: rigid-target docking. With this in mind, we used self-docking (to dock ligands into their own crystallographic structure) and cross-docking (to dock ligands in crystallographic structures that are bound to other ligands) to evaluate the ability of docking methods to reproduce crystallographic binding orientations at the top first scoring position. Two different docking software packages, Glide [18,19,20] and AutoDock [21], were employed for the tests.

## 2. Results

### 2.1. Self-Docking

It is common in medicinal chemistry literature to use rigid-target docking methods to explore the binding positions of novel synthesized ligands inside a known target. In order to carry out these tasks, an X-ray crystallographic structure of the target, which is often in presence of a previously reported ligand, is needed. When several crystallographic structures are present, one of them is selected. The next task is to demonstrate that the selected protein structure is reliable to accomplish docking experiments. A common test to justify the selection is to try to reproduce the orientation of the ligand that is inside the selected crystallographic structure: self-docking according to our definition in the Materials and Methods section.

**Figure 1:**
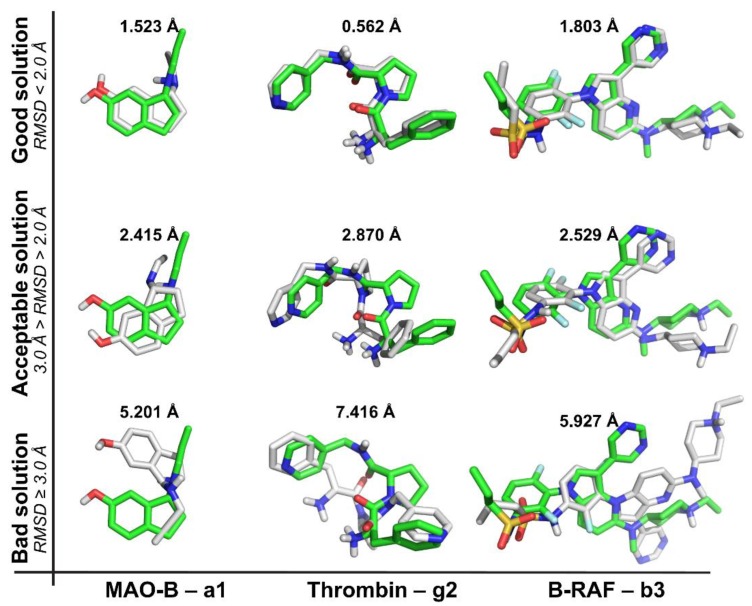
Examples of RMSD for docked ligands (gray) with respect to reference ligand at the crystal structures (green) for illustrating good (RMSD ≤ 2.0 Å), acceptable (RMSD > 2.0 Å and <3.0 Å) and bad (RMSD ≥ 3.0 Å) solutions in each target protein. **a1**: 5-hydroxy-*N*-propargyl-1(R)-aminoindan; **g2**: d-phenylalanyl-*N*-(pyridin-4-ylmethyl)-l-prolinamide; **b3**: *N*-(3-{5-[(1-ethylpiperidin-4-yl)(methyl)-amino]-3-(pyrimidin-5-yl)-1*H*-pyrrolo[3,2-b]pyridin-1-yl}-2,4-difluorophenyl)propane-1-sulfonamide.

In our experiment, each ligand from Table 1 was self-docked inside its own binding site. The top 10 poses (according to the scoring function energies) were selected to further structural analysis by calculating the root mean square deviation (RMSD) of each pose against its conformation in the crystal structure. We used three different RMSD classifications for docking solutions: (a) good solution when RMSD ≤ 2.0 Å [8,22], (b) acceptable solutions when RMSD is between 2.0 and 3.0 Å, and (c) bad solutions when RMSD ≥ 3.0 Å. For one ligand-protein pair, when a pose is classified as a good solution, this means that the scoring function reproduced the crystallographic binding orientation. To verify that RMSD classifications were adequate, compounds **a1**, **g2** and **b3** (Table 1) were docked in their respective targets (MAO-B, thrombin and B-RAF, respectively). Selected docking solutions are represented in Figure 1 (three of the ten poses are displayed) including good, acceptable and bad solutions according to above mentioned RMSD criteria. It is clear that an RMSD < 2.0 Å corresponds to good docking solutions. On the other hand, docking solutions with RMSD between 2.0 and 3.0 Å deviate from the position of the reference, but they keep the desired orientation. Finally, docking solutions with RMSD > 3.0 Å are completely wrong.

**Figure 2:**
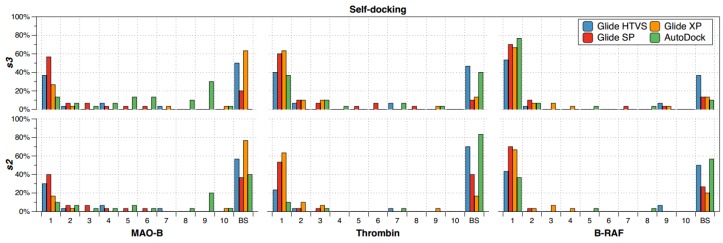
Self-docking percentages of recurrence of the best solution at each scoring position for the targets MAO-B, thrombin, and B-RAF considering *s2* (**bottom**) and *s3* (**top**) criteria. Glide HTVS (blue), Glide SP (red), Glide XP (orange) and AutoDock (green) were used as docking methods. Docking experiments were performed in triplicate.

The self-docking experiments were performed for the 30 different ligands from Table 1 into their own binding sites. Each docking was performed in triplicate to have a most representative exploration of the docking solutions. For each protein-ligand complex, the ten better obtained poses according to scoring functions (in Glide HTVS, Glide SP, Glide XP, and AutoDock methods) were analyzed by using the RMSD with respect to the reference complex. The best solution *s2* was defined for each complex (and for each docking method) as the docking solution with RMSD < 2.0 Å at the best scoring position. At the same time, the best solution *s3* was defined for each complex (and for each docking method) as the docking solution with RMSD < 3.0 Å at the best scoring position. *s2* only accounts for good solutions, while *s3* accounts for good or acceptable solutions. For each *s2* and *s3* values, the specific scoring position between 1 and 10 was annotated to construct a plot of the percentage of recurrence of the best solution at each scoring position for the targets MAO-B, thrombin and B-RAF; Figure 2 shows these plots for the used docking methods. For a protein-ligand complex, when none of the scoring positions contain a solution that complies with *s2* or *s3*, then it is annotated as a bad solution (BS), which indicates that there are no good poses for this case.

Self-docking results in Figure 2 allow the analysis of the recurrence of the best solution in the top first scoring position for different targets by using different docking methods. At first, it is clear that none of the docking methods obtained the best solution (under *s2* or *s3* criteria) at top first scoring position for all the ligands in interaction with the three targets under study. This means that it is not completely reliable to select the top first scoring solution in the simplest docking experiment: the self-docking.

If a rigorous criterion is selected, where the best solution *s2* is defined, the best solution in the top 1st scoring position have different recurrences according to the target and selected docking method. With regard to this, it is possible to extract some conclusions from the bottom part of the Figure 2:

(a) The recurrence of the top first scoring position was higher for B-RAF inhibitors by using all the docking methods.
(b) The recurrence of the top first scoring position was higher when the docking methods Glide SP and XP were used.
(c) In the self-docking study, Autodock was the worst method to get the best solution at the top first scoring position.

If the less rigorous criterion is selected, where the best solution *s3* is defined (Figure 2 top), there are no big changes in the analysis; except for the ability of AutoDock for detecting the best solutions at the top first scoring position for B-RAF inhibitors. This means that AutoDock yielded acceptable solutions for this specific target.

Other important point is the recurrence of BSs when self-docking is performed. When the best solution *s2* is defined (Figure 2 bottom), there are many BSs for different combinations of docking method with a specific target. For instance, there were many BSs when Glide XP was applied to study MAO-B inhibitors. This means that there were many MAO-B X-ray crystallographic structures that give no good solutions when the simplest self-docking was applied. It should be recalled that *s2* excludes acceptable solutions; therefore, when the best solution *s3* is defined for self-docking (Figure 2 top), there are less BSs.

Several lessons are provided from the self-docking experiment. The reliability on the top first scoring position as the best solution depends on the target under study and the docking method. In our study, B-RAF seems to be the more adequate target for self-docking; meanwhile, MAO-B seems to have difficulties during self-docking. On the other hand, Glide SP and XP seem to be the most adequate methods for self-docking. Noteworthy, it is no gain when the more precise XP version is used; in fact, SP is better for MAO-B and B-RAF inhibitors. 

When the best solution is not found at the top first scoring position, it is possible to find it at the second, third or other position, but, in several cases, there are no good solutions. It is not clear why several crystallographic structures fail during self-docking, and the consequences of that for a cross-docking experiment. However, it sounds logical and prudent not to take a structure for performing cross-docking if we know in advance that it failed when self-docking was done.

### 2.2. Cross-Docking

The most common practice in docking experiments is the inclusion of novel ligands inside a binding site occupied by a previous reported molecule: cross-docking according to our definition in Materials and Methods section. To test if cross-docking deteriorates the recurrence of the best solution in the top first scoring position for different targets by using different docking methods, we used the three compounds that yielded the best solutions in the self-docking experiments applied to each target for performing additional cross-docking experiments. The selected compounds were **b1**, **h1**, **j1** (MAO-B inhibitors), **b2**, **g2**, **h2** (thrombin inhibitors), **b3**, **f3**, and **g3** (B-RAF inhibitors). They were cross-docked against the remaining nine receptor crystal structures, and the obtained poses were compared with the conformation and orientation of the compound in the original crystal structure by means of RMSD measures.

Each cross-docking was performed in triplicate to have a most representative exploration of the docking solutions. For each protein-ligand complex, the ten better obtained poses according to scoring functions (in Glide HTVS, Glide SP, Glide XP and AutoDock methods) were analyzed by using the RMSD with respect to the reference complex. The best solutions *s2* were defined for each complex (and for each docking method) as in previous self-docking. 

**Figure 3:**
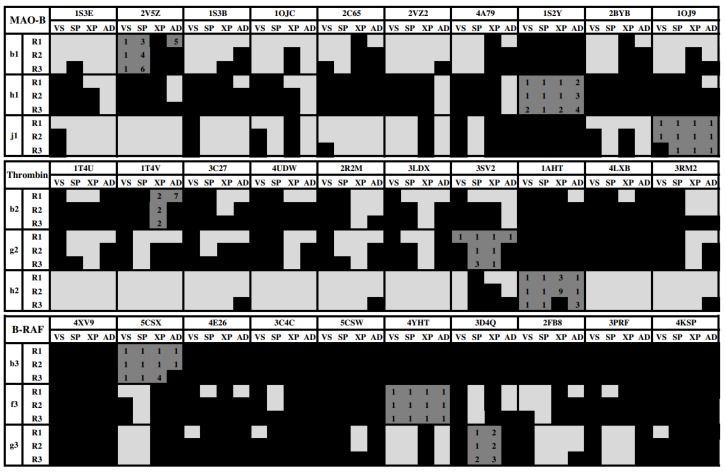
Matrix of good and bad solutions obtained by cross-docking ligands **b1**, **h1**, **j1** into MAO-B; **b2**, **g2**, **h2** into thrombin; and **b3**, **f3**, **g3** into B-RAF binding sites. Good solutions are defined considering RMSD under threshold of 2.0 Å (*s2* criterion). Light gray squares represent instances with at least one good solution was found, and black squares represent instances with only BSs. Dark gray squares represent self-docking, with numbers inside indicating the ranked poses.

Figure 3 shows the ability of the docking methods to find a good solution in cross-docking experiments inside the binding sites of the crystallographic structures of the studied targets. Dark gray squares represent self-docking, light gray squares represent instances with at least one good solution, and black squares represent instances with only BSs. The analysis reflects some interesting points:

(a) There are compounds that are well oriented in most of the crystal structures, but there are others that are conflictive. For instance, compound **h2** had good orientations in almost all the crystallographic structures of thrombin, but **b2** and **g2** cross-docking experiments failed in most of the instances.
(b) The selection of the docking method was crucial in some cases; for instance, **h1** had good orientations in almost all the crystallographic structures of MAO-B when AutoDock was used, but almost all cross-docking experiments for this compound failed when Glide HTVS, SP and XP were used.
(c) B-RAF was a conflictive target for cross-docking. All its inhibitors were bad oriented in almost all crystallographic structures by using all docking methods. Particularly, compound **b3** failed in all the instances.

**Figure 4:**
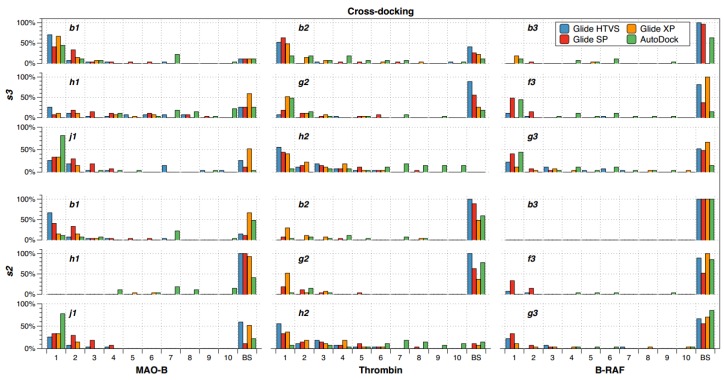
Cross-docking percentages of recurrence of the best solution at each scoring position for the targets MAO-B, thrombin and B-RAF considering *s2* (**bottom**) and *s3* (**top**) criteria. Glide HTVS (blue), Glide SP (red), Glide XP (orange) and AutoDock (green) were used as docking methods. Docking experiments were performed in triplicate.

The recurrence of the best solution at the first scoring position was also analyzed for cross-docking experiments. For this, the specific scoring positions between 1 and 10 (considering *s2* and *s3* criteria) were annotated to construct a plot of the percentage of recurrence of the best solution at each scoring position for the targets MAO-B, thrombin, and B-RAF; Figure 4 shows these plots for the used docking methods. BSs were also annotated for the cases that have no good poses. 

Once again, it is clear that none of the docking methods obtained the best solution during cross-docking experiments (under *s2* or *s3* criteria) at top first scoring position for all the ligands in interaction with the three targets under study. This means that it is not completely reliable to select the top first scoring solution when cross-docking is achieved without an additional analysis. If a rigorous criterion is selected, when the best solution *s2* is defined, the best solution at the top 1st scoring position have different recurrences according to the compound, target and selected docking method. With regard to this, it is possible to conclude from the bottom part of the Figure 4 that the recurrence of the top first scoring position was higher for a few combinations of compound and docking method. For instance, the MAO-B inhibitors **b1** and **j1** had around 65% and 80% of recurrences of the top first scoring position when Glide HTVS and AutoDock are used, respectively. On the other hand, the thrombin inhibitors **g2** and **h2** had around 50% of recurrences of the top first scoring position when Glide XP and Glide HTVS are used, respectively. The remaining cases had small recurrences of the top first scoring position. In general, it is possible to see that cross-docking was characterized by a poor recurrence of the top first scoring position and many BSs. It suggests that it is not reliable to select the top first scoring position as the best solution when cross-docking is performed. 

If the less rigorous criterion is selected, where the best solution *s3* is defined (Figure 4 top), there are subtle changes in the analysis. More combinations of compound and docking method yielded the best solution at the top first scoring position; however, it is necessary to remember that this improvement is due to the addition of acceptable cases with no optimal orientation in many cases. 

Another important point is the recurrence of BSs when cross-docking is performed. When the best solution *s2* is defined (Figure 4 bottom), there are many BSs for different combinations of compounds with docking methods. For instance, there were many BSs when all the docking methods were applied to study the three B-RAF inhibitors. This suggests that a special care should be taken when cross-docking is performed for novel B-RAF inhibitors. On the other hand, the number of BSs is low for the thrombin inhibitor **h2** by using all the docking methods. This suggests that there are compounds more likely to be well oriented inside different conformations of the binding site. The inclusion of acceptable solutions (*s3* criterion) reduces the number of BSs (Figure 4 top), at the expense of lower quality of the orientations.

Several lessons are provided from the cross-docking experiment. We observed for self-docking that the reliability on the top first scoring position as the best solution depends on the target under study and the docking method, but further experiments show that it is not possible to rely on the top first scoring when cross-docking is done. Previously, we observed that B-RAF seemed to be the more adequate target for self-docking; but further experiments show that B-RAF is the less adequate target when cross-docking was done. MAO-B had difficulties during self-docking, but it was the target with a better performance during cross-docking. 

The main reason of the success rate reduction for cross-docking method is that, even though the structures correspond to the same protein (MAO-B, thrombin or B-RAF in our work), the global shape of the binding site varies for different crystal structures of the same protein due to different orientations of the side chains of several residues. To observe the principal differences between different structures of the same protein, the crystallographic MAO-B, thrombin and B-RAF structures were aligned, and the RMSD values for comparison between residues were calculated to study how different are the binding sites (results are represented in the Electronic Supplementary Material, Figures S1–S3). In general, RMSD values were below 0.5 Å for MAO-B and thrombin structures, and were above 1.0 or 1.5 Å for B-RAF in most of the structural alignments.

We selected the residues that displayed different conformations among the studied crystallographic structures due to the presence of different ligands and compared their RMSD values. In MAO-B crystal structures, the residues with higher RMSD values were F103, I199 and Y326 (Supplementary Material, Figures S1B–D); however, values below 2 Å were observed for I199 and Y326 because a small displacement of side chains of these residues in the structure with PDB code 1S2Y. Additionally, values below 3 Å were observed for F103 because higher displacements of its side chain in the structures with PDB codes 1S2Y and 4A79. These observations explain the bad performance of the ligand **h1** and the protein structure with PDB code 1S2Y during the rigid cross-docking.

In thrombin crystal structures, the residues with higher RMSD values were W86 and E232 (Supplementary Material, Figures S2B–D); however, values below 1.5 Å were observed for W86 because a small displacement of its side chain in the structure with PDB code 4LXB. Additionally, values below 3 Å were observed for E232 because higher displacements of its side chain in the structures with PDB codes 3SV2, 1AHT, and 4UDW. These observations explain the bad performance of the protein structure with PDB code 1AHT during the cross-docking of ligands **b2** and **g2**. Despite the displacement of the side chain of E232 in the structure with code 1AHT, the ligand in this structure (**h2**) had good performance when it is cross-docked in other structures, indicating that displacements do not always negatively affect cross-docking. 

Finally, in B-RAF crystal structures we found the largest numbers of residues with high RMSD values: I463, K483, E501, L505, and F583 (Supplementary Material, Figures S3B–D). Values below 2.5 Å were observed for K483 and L505 due to displacements of their side chains when the crystallographic structures were compared. High RMSD values were also observed for I463 because its longer displacement in the structures with PDB codes 3D4Q and 3PRF. This residue is located at the glycine-rich loop of B-RAF, which present backbone conformational changes for adapting to ATP or inhibitor binding [14,46]. The largest displacements in B-RAF were observed for E501 (backbone and side-chain) and F583 (side-chain) with RMSD values above 4 Å for their side chains when comparing different crystallographic structures. E501 is the conserved glutamate at the αC-helix; this helix has a high mobility involved in controlling the dynamic equilibrium between active and inactive functional forms of protein kinases. F583 is part of the hydrophobic wall of the ATP binding site which adapts side chain orientation to different inhibitors. These observations reveal that B-RAF binding site changes more than the ones for MAO-B and thrombin; this explains the bad results of B-RAF in cross-docking. 

Additional information was obtained when various characteristics of the protein binding sites and ligands were analyzed for each MAO-B, thrombin, and B-RAF PDB structures. Protein binding site volumes (BSV), volume depth (VD) values, and averaged VDs were obtained for the protein binding sites by using the software POCASA [47]. VD is the volume depth value, which is determined by summing the depth of all pocket points, where the depth of every pocket point is defined as the shortest distance from pocket point to probe surface. Average VD is the average of the depth of every pocket points. Binding site volumes were calculated considering a probe radius of the probe sphere of 1 Å, single point flag of 12, protein depth flag of 18, and grid size of 1 Å [47]. Number of rotatable bonds and molecular weights of the inhibitors inside each target were calculated by using Molinspiration web (http://www.molinspiration.com/cgi-bin/properties). The calculated characteristics for protein binding sites and ligands are reported in Table 2. 

MAO-B has a small binding site (low BSV) in a deeper zone of the protein (high VD and averaged VD). There are big differences in BSVs among different PBD structures (BSDs are 97 and 289 Å^3^ for PDB structures 4A79 and 1OJ9, respectively), which could lead to difficulties in cross-docking experiments. However, analysis of the Figure 3 does not give evidence that the differences in MAO-B BSVs caused the failures in cross-docking. Major problems were found for compound **h1** (from PDB 1S2Y), which has the lower molecular weight and only two rotatable bonds. Compounds **b1** and **j1** had major problems in the PDB structure where **h1** is bound: 1S2Y. While **b1** and **j1** are bigger ligands with more rotatable bonds, they have good solutions in other MAO-B structures with smaller BSVs (for instance, in PDB 2VZ2). Therefore, we consider that the problem in cross-docking for the studied MAO-B inhibitors is mainly due to orientations of the residue side chains in the binding site (as we observed previously in Figure S1 of the Supplementary Material), instead of BSVs or ligand characteristics.

Thrombin has a bigger binding site (BSV above 280 Å^3^ in most of PDB structures) at the surface of the protein (low VD and averaged VD). BSVs are similar, being between 280 and 370 Å^3^ in the majority of the PDB structures, with the only exception of the PDB structure 4UDW. Thrombin inhibitors have many rotatable bonds, with the exception of compound **h2**. It is just this compound the best one in cross-docking experiments, while compounds **b2** and **g2** (with 10 and six rotatable bonds respectively) fail in most of cross-docking experiments. Therefore, we consider that the problem in cross-docking for the studied thrombin inhibitors is mainly due to the number of rotatable bonds of the ligands, instead of different BSVs or different orientations of the residue side chains in the thrombin binding site. 

B-RAF has three size ranges for the binding site. The smaller range has BSVs between 180 and 220 Å^3^ (PDB structures 5CSW, 4YHT, 3D4Q, 2FB8, and 3PRF), the intermediate range has BSVs between 310 and 360 Å^3^ (PDB structures 4XV9, 4E26, and 3C4C), and the bigger range has BSVs around 440 Å^3^ (PDB structures 5CSX and 4KSP). VD and averaged VD values indicate that B-RAF binding site is not too deep from the surface. The big differences in BSVs among different PBD structures could explain the observed difficulties in B-RAF cross-docking experiments. B-RAF inhibitor **b3** has many rotatable bonds and was bound to one of the biggest binding sites (PDB: 5CSX, BSV = 438 Å^3^). It had no solutions when it was cross-docked in the other binding sites with lower or similar BSVs. Compounds **f3** and **g3** have less rotatable bonds and were bound to smaller size binding sites. In general, they also fail in most of the cross-docking experiments, but they have solutions mainly when cross-docking was done inside other PDB structures in the same BSV range, such as 4YHT, 3D4Q, 2FB8, and 3PRF. This analysis suggests that the number of rotatable bonds and the different BSVs are the key effects that lead to problems in B-RAF cross-docking experiments. At the same time, different orientations of the residue side chains in the binding site (as we observed previously in Figure S3 of the Supplementary Material) cause the differences in B-RAF BSVs.

## 3. Discussion and Recommendations

Here, we must point out that cross-docking is the most common practice in docking. According to our definition (in the Materials and Methods section), when a crystallographic structure is used to perform docking of a novel ligand (a different one to what is found in the original structure), we have a cross-docking experiment, but the most serious problem is that the novel ligands typically have no reference structure to help in the selection of the best solution. Therefore, if top first scoring position cannot be relied upon to select the best docking solution, other criteria should be taken into account.

In the work of Sutherland et al. assessing cross-docking accuracy [6], the authors found that using the protein structure from the complex that contains the bound ligand most similar to the docked ligand increased docking accuracy for all methods (defined as “similarity selection”). Another interesting point authors found is that there is no relationship between resolution and cross-docking accuracy. Therefore, the common practice of choosing structures for docking based on their resolution appears to be unfounded. It is well known that the predictive power of a docking method depends on several factors connected with the docking program used as well as on the studied system; no general rules of thumb exist. Verkhivker et al. [48] distinguished docking hard failures when the crystallographic pose cannot be sampled and soft failures when the crystallographic pose can be sampled but is not the top scoring one. They considered that soft failures in self-docking are mainly due to limits in the scoring functions and scoring schemes.

According to our experience, an option is the use of previous literature and crystallographic information to define constraints to the solution. These constraints, stated as Essential Chemical Interactions Described for Analogue Ligands (ECIDALs) [49], contain the chemical interactions that seem to be essential after a comprehensive inspection of the crystallographic data available in PDB, considering the target under study or other proteins from the same family. Under this strategy, the best solution of a cross-docking must be selected by considering the most negative scoring energy which complies with ECIDALs.

It is important to discuss about the rigidity of the binding site in the receptor during cross-docking. The standard cross-docking practice considers that ligands are docked into a rigid receptor, although this assumption could lead to misleading results. However, it is known that many proteins undergo side-chain as well as backbone movements, upon ligand binding. The possibility to consider these movements is the application of flexibility to residues in the binding site of the protein during docking. This methodology is referred to as induced fit docking and allows the receptor to alter the shape of the active site and BSV due to conformational changes induced by the bound ligand [50]. A more realistic description of the complex can be obtained by using this method, but the movement of the residues lead to a very computationally expensive process; therefore, a more reasonable strategy is the selection of a few flexible residues; but after this selection, the consideration of the ECIDALs is also needed to select the best solution.

## 4. Materials and Methods

### 4.1. Self-Docking and Cross-Docking

Let us check definitions of self-docking and cross-docking in this work. For a unique target protein A, we selected the crystallographic structure of a protein-ligand complex $A_{\alpha }\alpha$ deposited in Protein Data Bank (PDB), where $A_{\alpha }$ represents the receptor with the optimal conformation to bound the ligand α in its active site. Meanwhile, α is the ligand contained in this crystallographic structure. The self-docking is the process of docking α inside $A_{\alpha }$ (Equation (1)). This process should be simpler since $A_{\alpha }$ binding site have a contoured shaping for perfect fit of α and the residues in the binding site are present in conformations that allow establishing all the chemical interactions without needing large scale movements of their side chains:

(1) $$A_{\alpha }{}_{\left(Receptor\right)}+\;\alpha_{\left(Ligand\right)}=A_{\alpha }\alpha_{\left(Complex\right)}$$

When docking methodology is employed to dock the ligand α into the receptor $A_{\alpha }$ we assume this practice as self-docking. For the same target protein, we can select other crystallographic structure $A_{\beta }\beta$ deposited in PDB, where $A_{\beta }$ represents the same receptor with the optimal conformation to bound the ligand β in its active site. Self-docking can be applied to dock the ligand β into the receptor $A_{\beta }$. However, when the ligand β is docked into a different crystallographic structure of the same receptor (for instance $A_{\alpha }$) we assume this practice as cross-docking. The last process is represented in Equation (2):

(2) $$A_{\alpha }{}_{\left(Receptor\right)}+\;\beta_{\left(Ligand\right)}=A_{\alpha }\beta_{\left(Complex\right)}$$

In this process, it is important to consider that $A_{\alpha }$ binding site does not necessarily have a contoured shaping for perfect fit of β since the residues in its binding site are present in conformations that maybe need large scale movements of their side chains for establishing the optimal chemical interactions. It is necessary to highlight that the last process, cross-docking, is the habitual goal in docking experiments, where a ligand have to be docked in a protein which is complexed with a molecule different from the one used for the docking.

### 4.2. Protein Targets under Study

Three different target proteins were selected: monoamine oxidase B (MAO-B), thrombin and B-RAF protein kinase. These proteins are co-crystallized with several inhibitors. Ten different protein-inhibitor crystallographic structures were downloaded from the PDB repository for each target protein under study (PDB codes are in Table 1). MAO-B is an isoform of monoamine oxidase that is involved in dopamine metabolism [51]. MAO-B inhibitors prevent the breakdown of dopamine in the brain, thereby increasing dopamine levels. They are in clinical use for the treatment of Parkinson’s disease, and have also been investigated for potential neuroprotective benefits [52,53]. Thrombin is a serine protease with a central role in thrombosis and haemostasis, which makes it an attractive target for antithrombotic therapy [54]. Thrombin inhibitors are designed for anticoagulation therapy, where they have more predictable anticoagulant effects compared with traditional anticoagulants, such as heparins, because of their lack of binding to other plasma proteins [55], anti-platelet effects [56], and no heparin-induced thrombocytopenia effects [57]. B-RAF protein kinase is one of the Raf protein kinases, which has been identified as the primary MEK activator in the Ras-Raf-MEK-ERK signaling pathway [58]. It is one of the most frequently mutated genes in human cancers [59]; therefore, therapeutic inhibition of oncogenic B-Raf kinase activity is considered a promising strategy to treat cancer [60]. All targets were processed with the Protein Preparation Wizard in the Schrödinger Suite (Maestro 9.0, 2007; Schrödinger, LLC: New York, NY, USA). Hydrogen atoms were added followed by the adjustment of bond orders. The protonation and tautomeric states for protonable residues were adjusted to match pH = 7.4. Missing residues and loop segments near the active site were added by using Prime (Prime 2.1, 2009; Schrödinger, LLC). Water molecules were deleted. Proteins were finally subjected to geometry optimization by using OPLS_2005 force field [61].

### 4.3. Docking Experiments

Two experiments were applied for each target:

(a) Self-docking of each ligand inside its own protein structural conformation.
(b) Cross-docking of three selected ligands inside the remaining nine protein structures.

All the studied ligands in this work were prepared using LigPrep with the force field OPLS_2005 [61]. Docking tests were performed using the softwares Glide and Autodock. Glide offers a complete solution for ligand–receptor docking and is widely used for drug discovery [62,63], virtual screening [64,65], structure-activity relationship analysis [9,66], etc. Grid boxes for self-docking and cross-docking experiments were centered in the ligand position coming from the crystal structures. The grid boxes’ dimensions were (20 × 20 × 20) Å^3^ in order to include the whole binding site for the three targets under study. High-throughput virtual screening (HTVS), standard precision (SP), and extra precision (XP) Glide modes were proved. Default docking parameters were used. Glide docking uses hierarchical filters to find the best ligand binding locations in the defined receptor grid space. The filters include positional, conformational, and orientational sampling of the ligand and subsequent energy evaluation of the interactions between the ligand and the protein [18,19,20]. Ligand minimization in the receptor field is carried out using the OPLS-AA force field [67] with a distance-dependent dielectric of 2.0. Afterward, the lowest energy poses are subjected to a Monte Carlo (MC) procedure that samples the nearby torsional minima. The best poses for a given ligand are determined by the GlideScore score [68], including terms for buried polar groups and steric clashes.

Autodock parameters were defined in a similar way as in Glide, with grid boxes dimensions of 20 × 20 × 20 Å^3^. AutoDock uses a semi-empirical free energy force field to predict binding energies or ligands to macromolecular targets and Lamarckian genetic algorithm (GA) to search for docking solutions [69]. The force field is based on a comprehensive thermodynamic model that allows incorporation of intramolecular energies into the predicted free energy of binding [70]. Each ligand was located in the grid box for each docking run and torsional degrees of freedom were defined. The GA was applied with an initial population of 100 randomly placed individuals, a maximum number of 1 × 10^6^ energy evaluations, a maximum number of 3 × 10^4^ generations, a mutation rate of 0.02, a crossover rate of 0.80, and an elitism value of 2.

Each self-docking or cross-docking experiment between a target protein (MAO-B, thrombin or B-RAF) and a ligand was repeated three times, accounting for replicated instances. The effectiveness of the docking experiment (self-docking or cross-docking) in reproducing the crystallographic binding orientation of a ligand α was determined by comparing the docked pose with the orientation of the ligand in its native crystallographic structure $A_{\alpha }$. In the case of MAO-B, the cofactor FAD was present in the binding site during docking experiments. The RMSD was employed as the term for performing such comparison, where ligand atoms were matched one to one and symmetrical atoms were considered equivalent. For comparing cross-docking poses, receptor structures were superimposed using C***α*** of binding site amino acids, by considering that amino acids that surround 10 Å the ligand in its native crystallographic structure form the binding site.

## 5. Conclusions

We presented experiments using self and cross-docking methodologies applied to three biological targets (MAO-B, thrombin and B-RAF) to analyze the recurrence of the best solution at the top first scoring position. We used ten crystal structures for each target protein and the softwares Glide (HTVS, SP and XP) and AutoDock. In general, the docking methods proved to efficiently reproduce crystallographic binding orientations when self-docking was performed. However, we found that right predictions of the binding orientations at the top first scoring position depend on the target under study, the ligand, and the software.

To approach our experiment to the real docking practice, since users usually dock ligands into receptor crystal structures fitted to a different ligand, we performed cross-docking by exploring three of the studied ligands inside the remaining crystallographic structures. Results suggest that each studying system is independent and must be carefully treated for cross-docking simulations. We found that the success rates in reproducing the known bound conformation of the ligand at the top first scoring position was significantly lower in cross-docking than in self-docking because the binding site of each receptor is fitted to its own ligand.

Information in this manuscript orient researchers to successfully employ docking methodology for selecting the best orientation of ligands into a receptor target. Docking methods give an estimation of a scoring function to select the best solution; however, it is not recommendable to give the best score (the lower energy value) as the final solution without further analysis. Previous literature has suggested that using the protein structure from the complex that contains the bound ligand most similar to the docked ligand (“similarity selection”) increases cross-docking accuracy. On the other hand, many authors have proposed the selection of a small set of representative docking poses forming clusters to get a reliable solution. In our experience, the use of the literature and structural information contained in PDB helps to define the ECIDALs of the target. In that regard, the selection of the best scoring solution that complies with the defined ECIDALs is a rational approach to get a reliable solution.

## Figures and Tables

**Table 1:** Crystal structures selected for self-docking and cross-docking experiments.

		MAO-B	
PDB ID	Resolution (Å)	Ligand	Reference
1S3E	1.60	**a1: 5-hydroxy-*N*-propargyl-1(R)-aminoindan**	[23]
2V5Z	1.60	**b1:** Safinamide	[24]
1S3B	1.65	**c1:***N*-methyl-*N*-propargyl-1(R)-aminoindan	[23]
1OJC	2.40	**d1:** 4-chloro-*N*-(2-hydrixyethyl)benzamide	[25]
2C65	1.70	**e1:** (1R)-4-({[ethyl(methyl)amino]carbonyl}oxy)-*N*-methyl-*N*-[(1E)-prop-2-en-1-ylidene]undan-1-aminium	[26]
2VZ2	2.30	**f1:** (1Z)-4-(4-fluorophenyl)-2-methylidenebutan-1-imine	[27]
4A79	1.89	**g1:** Pioglitazone	[28]
1S2Y	2.12	**h1: *N*-propargyl-1(S)-aminoindan**	[23]
2BYB	2.20	**i1:***N*-methyl-*N*-[(1R)-1-methyl-2-phenylethyl]prop-2-en-1-amine	[29]
1OJ9	2.30	**j1: 1,4-duphenyl-2-butene**	[25]
		**Thrombin**	
**PDB ID**	**Resolution (Å)**	**Ligand**	**Reference**
1T4U	2.00	**a2:** 2-methanesulfonyl-benzenesulfonic acid 3-methyl-5-((1-amidinoaminooxymethyl-cyclopropyl)methyloxy)-phenylester	[30]
1T4V	2.00	**b2: *N*-allyl-5-amidinoaminooxy-propyloxy-3-chloro-*N*-cyclopentylbenzamide**	[30]
3C27	2.18	**c2:***N*-[2-(carbamimidamidooxy)ethyl]-2-{6-cyano-3-[(2,2-difluoro-2-pyridin-2-ylethyl)amino]-2-fluorophenyl}acetamide	
4UDW	1.16	**d2:**d-phenylalanyl-*N*-(2,5-dichlorobenzyl)-l-prolinamide	[31]
2R2M	2.10	**e2:***N*-[2-({[amino(imino)methyl]amino}oxy)ethyl]-2-{6-chloro-3-[(2,2-difluoro-2-phenylethyl)amino]-2-fluorophenyl}acetamide	[32]
3LDX	2.25	**f2:** RWJ-671818	
3SV2	1.30	**g2: d-phenylalanyl-*N*-(pyridin-4-ylmethyl)-l-prolinamide**	[33]
1AHT	1.60	**h2: (2S)-3-(4-carbamimidoylphenyl)-2-hydroxypropanoic acid**	[34]
4LXB	1.61	**i2:** 5-chloro-thiophene-2-carboxylic acid [(S)-2-[2-difluoromethoxy-3-(2-oxo-piperidin-1-yl)-benzenesulfonylamino]-3-((S)-3-dimethylamino-pyrrolidin-1-yl)-3-oxo-propyl]-amide	[35]
3RM2	1.23	**j2:***N*-(benzylsulfonyl)-3-cyclohexyl-d-alanyl-*N*-(4-carbamimidoylbenzyl)-l-prolinamide	[36]
		**B-RAF protein kinase**	
**PDB ID**	**Resolution (Å)**	**Ligand**	**Reference**
4XV9	2.00	**a3:***N*-{3-[(5-chloro-1*H*-pyrrolo[2,3-b]pyridin-3-yl)carbonyl]-2,4-difluorophenyl}-4-(trifluoromethyl)benzenesulfonamide	[37]
5CSX	2.51	**b3: *N*-(3-{5-[(1-ethylpiperidin-4-yl)(methyl)amino]-3-(pyrimidin-5-yl)-1*H*-pyrrolo[3,2-b]pyridin-1-yl}-2,4-difluorophenyl)propane-1-sulfonamide**	[38]
4E26	2.55	**c3:** 5-chloro-7-[(R)-furan-2-yl(pyridin-2-ylamino)methyl]quinolin-8-ol	[39]
3C4C	2.57	**d3:***N*-{3-[(5-chloro-1*H*-pyrrolo[2,3-b]pyridin-3-yl)carbonyl]-2,4-difluorophenyl}propane-1-sulfonamide	[40]
5CSW	2.66	**e3:** Dabrafenib	[38]
4YHT	3.05	**f3: 3-[(5-chloro-7*H*-pyrrolo[2,3-d]pyrimidin-4-yl)amino]-*N*-methyl-4-(morpholin-4-yl)benzenesulfonamide**	[41]
3D4Q	2.80	**g3: (1E)-5-(1-piperidin-4-yl-3-pyridin-4-yl-1*H*-pyrazol-4-yl)-2,3-dihydro-1*H*-inden-1-one oxime**	[42]
2FB8	2.90	**h3:** (1Z)-5-(2-{4-[2-(dimethylamino)ethoxy]phenyl}-5-pyridin-4-yl-1*H*-imidazol-4-yl)indan-1-one oxime	[43]
3PRF	2.90	**i3:** 2-chloro-5-{[2-(pyrimidin-2-yl)furo[2,3-c]pyridin-3-yl]amino}phenol	[44]
4KSP	2.93	**j3:***N*-{7-cyano-6-[4-fluoro-3-({[3-(trifluoromethyl)phenyl]acetyl}amino)phenoxy]-1,3-benzothiazol-2-yl}cyclopropanecarboxamide	[45]

Note: Ligands used in cross-docking experiments are in bold letter.

**Table 2:** Characteristics of the protein binding sites and ligands for each PDB structure under study.

			Protein Binding Site		Ligand	
PDB	Ligand	BSV (Å^3^) ^a^	VD Value ^b^	Averaged VD ^c^	Number of Rotatable Angles	MW (g/mol)
MAO-B						
1S3E	**a1**	221	2485	11.2474	2	187.2
2V5Z	**b1**	237	3162	13.3418	6	300.3
1S3B	**c1**	110	1417	12.8848	2	201.2
1OJC	**d1**	270	2900	10.7420	3	199.6
2C65	**e1**	276	2974	10.7754	5	287.3
2VZ2	**f1**	116	1555	13.4080	4	177.2
4A79	**g1**	97	1274	13.1409	7	356.4
1S2Y	**h1**	132	1677	12.7071	2	171.2
2BYB	**i1**	286	3097	10.8298	5	189.3
1OJ9	**j1**	289	3285	11.3691	4	208.3
Thrombin						
1T4U	**a2**	286	670	2.34499	10	483.5
1T4V	**b2**	388	1054	2.71649	10	394.9
3C27	**c2**	366	937	2.56011	10	435.4
4UDW	**d2**	172	486	2.82946	6	420.3
2R2M	**e2**	333	796	2.39239	10	443.8
3LDX	**f2**	336	805	2.39583	10	423.4
3SV2	**g2**	297	835	2.81145	6	352.4
1AHT	**h2**	321	852	2.65628	4	208.2
4LXB	**i2**	334	790	2.36727	11	648.1
3RM2	**j2**	308	724	2.35281	11	553.7
B-RAF						
4XV9	**a3**	359	1432	3.98886	6	515.8
5CSX	**b3**	438	2834	6.47108	9	569.6
4E26	**c3**	316	1030	3.26160	4	351.7
3C4C	**d3**	311	925	2.97428	6	413.8
5CSW	**e3**	180	909	5.05000	6	519.5
4YHT	**f3**	185	662	3.58198	5	422.9
3D4Q	**g3**	216	815	3.77623	3	377.4
2FB8	**h3**	202	708	3.50495	8	453.5
3PRF	**i3**	211	687	3.25750	2	338.7
4KSP	**j3**	444	1415	3.18769	9	556.5

^a^ BSV is the binding site volumen. ^b^ VD is the volume depth value, which is determined by summing the depth of all pocket points; the depth of every pocket point is defined as the shortest distance from pocket point to probe surface. ^c^ Averaged VD is the average of the depth of every pocket points. More information in reference [47].

## Supplementary Materials

The following are available online. Figure S1. RMSD values (in Å) of different MAO-B crystallographic structures (A), and of their residues F103 (B), I199 (C) and Y326 (D). Conformations of residues are represented to the right. Figure S2. RMSD values (in Å) of different thrombin crystallographic structures (A), and of their residues W86 (B) and E232 (D). Conformations of residues are represented to the right. Figure S3. RMSD values (in Å) of different B-RAF crystallographic structures (A), and of their residues I463 (B), K483 (C), E501 (D), L505 (E) and F583 (F). Conformations of residues are represented to the right.
