# Supplementary material for: Is It Reliable to Take the Molecular Docking Top Scoring Position as the Best Solution without Considering Available Structural Data?
Source: Molecules. 2018 Apr 28;23(5):1038. doi: 10.3390/molecules23051038 (PMC6102569; doi:10.3390/molecules23051038)
Supplement: Supplementary file 1 [file molecules-23-01038-s001.pdf]

Supplementary material

Is It Reliable to Take the Molecular Docking Top Scoring Position as the Best Solution without Considering Available Structural Data?

David Ramírez [1] and Julio Caballero [2,*]

[1] Instituto de Ciencias Biomédicas, Universidad Autónoma de Chile, 5 Poniente No. 1670, 3460000, Talca, Chile, david.ramirez@uautonoma.cl

[2] Centro de Bioinformática y Simulación Molecular (CBSM), Universidad de Talca. 1 Poniente No. 1141, 3460000, Talca, Chile.

* Correspondence: jcaballero@utalca.cl; Tel.: +56-712-418-850

A — MAO-B

	1S3E	2V5Z	1S3B	1OJC	2C65	2VZ2	4A79	1S2Y	2BYB	1OJ9
1S3E	0.00									
2V5Z	0.11	0.00								
1S3B	0.15	0.20	0.00							
1OJC	0.13	0.18	0.17	0.00						
2C65	0.17	0.21	0.17	0.23	0.00					
2VZ2	0.19	0.21	0.22	0.21	0.23	0.00				
4A79	0.14	0.16	0.20	0.16	0.25	0.20	0.00			
1S2Y	0.29	0.32	0.27	0.30	0.33	0.33	0.33	0.00		
2BYB	0.20	0.22	0.24	0.21	0.29	0.26	0.24	0.30	0.00	
1OJ9	0.13	0.15	0.19	0.12	0.22	0.20	0.17	0.32	0.22	0.00

B — F103

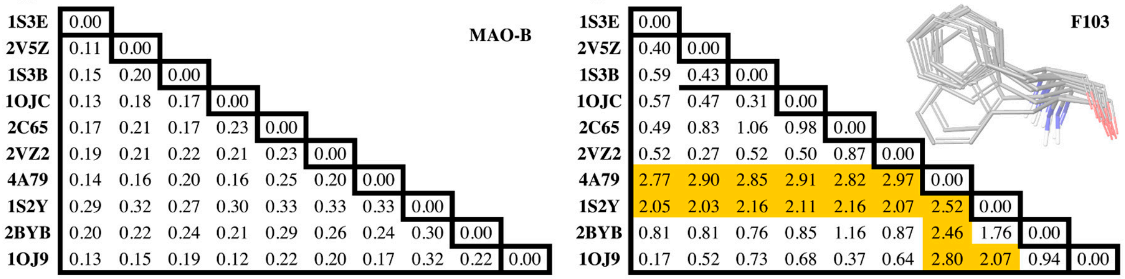

	1S3E	2V5Z	1S3B	1OJC	2C65	2VZ2	4A79	1S2Y	2BYB	1OJ9
1S3E	0.00									
2V5Z	0.40	0.00								
1S3B	0.59	0.43	0.00							
1OJC	0.57	0.47	0.31	0.00						
2C65	0.49	0.83	1.06	0.98	0.00					
2VZ2	0.52	0.27	0.52	0.50	0.87	0.00				
4A79	2.77	2.90	2.85	2.91	2.82	2.97	0.00			
1S2Y	2.05	2.03	2.16	2.11	2.16	2.07	2.52	0.00		
2BYB	0.81	0.81	0.76	0.85	1.16	0.87	2.46	1.76	0.00	
1OJ9	0.17	0.52	0.73	0.68	0.37	0.64	2.80	2.07	0.94	0.00

C — I199

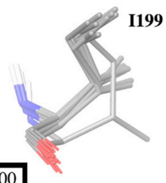

	1S3E	2V5Z	1S3B	1OJC	2C65	2VZ2	4A79	1S2Y	2BYB	1OJ9
1S3E	0.00									
2V5Z	0.55	0.00								
1S3B	0.10	0.59	0.00							
1OJC	0.83	0.67	0.84	0.00						
2C65	0.15	0.62	0.17	0.91	0.00					
2VZ2	0.60	0.35	0.62	0.59	0.72	0.00				
4A79	0.51	0.27	0.56	0.69	0.60	0.43	0.00			
1S2Y	1.75	1.72	1.72	1.39	1.82	1.50	1.74	0.00		
2BYB	0.42	0.69	0.41	0.93	0.40	0.79	0.60	1.93	0.00	
1OJ9	0.30	0.33	0.36	0.73	0.40	0.44	0.29	1.71	0.55	0.00

D — Y326

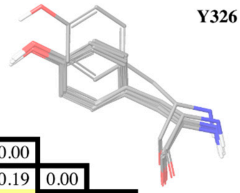

	1S3E	2V5Z	1S3B	1OJC	2C65	2VZ2	4A79	1S2Y	2BYB	1OJ9
1S3E	0.00									
2V5Z	0.12	0.00								
1S3B	0.09	0.16	0.00							
1OJC	0.19	0.15	0.19	0.00						
2C65	0.19	0.11	0.22	0.15	0.00					
2VZ2	0.33	0.24	0.34	0.17	0.22	0.00				
4A79	0.33	0.23	0.37	0.23	0.18	0.19	0.00			
1S2Y	1.34	1.44	1.32	1.46	1.52	1.59	1.64	0.00		
2BYB	0.25	0.28	0.30	0.32	0.31	0.43	0.40	1.42	0.00	
1OJ9	0.10	0.06	0.14	0.16	0.16	0.26	0.26	1.40	0.28	0.00

RMSD < 1	1 < RMSD < 2	2 < RMSD < 3	3 < RMSD < 4	4 < RMSD < 5	RMSD > 5

Figure S1. RMSD values (in Å) of different MAO-B crystallographic structures (A), and of their residues F103 (B), I199 (C) and Y326 (D). Conformations of residues are represented to the right.

A — Thrombin

	1T4U	1T4V	3C27	4UDW	2R2M	3LDX	3SV2	1AHT	4LXB	3RM2
**1T4U**	0.00									
**1T4V**	0.16	0.00								
**3C27**	0.24	0.21	0.00							
**4UDW**	0.34	0.33	0.37	0.00						
**2R2M**	0.22	0.17	0.17	0.35	0.00					
**3LDX**	0.22	0.18	0.20	0.35	0.15	0.00				
**3SV2**	0.24	0.21	0.23	0.24	0.21	0.23	0.00			
**1AHT**	0.23	0.20	0.23	0.35	0.20	0.21	0.24	0.00		
**4LXB**	0.26	0.24	0.24	0.27	0.24	0.25	0.17	0.25	0.00	
**3RM2**	0.26	0.24	0.29	0.25	0.25	0.26	0.11	0.24	0.19	0.00

B — W86

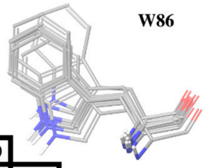

	1T4U	1T4V	3C27	4UDW	2R2M	3LDX	3SV2	1AHT	4LXB	3RM2
**1T4U**	0.00									
**1T4V**	0.37	0.00								
**3C27**	0.39	0.36	0.00							
**4UDW**	0.84	0.55	0.73	0.00						
**2R2M**	0.34	0.19	0.20	0.59	0.00					
**3LDX**	0.47	0.31	0.21	0.66	0.20	0.00				
**3SV2**	0.45	0.18	0.38	0.43	0.21	0.35	0.00			
**1AHT**	0.61	0.81	0.66	1.24	0.73	0.79	0.84	0.00		
**4LXB**	1.42	1.22	1.17	1.00	1.16	1.17	1.08	1.41	0.00	
**3RM2**	0.45	0.24	0.36	0.62	0.24	0.25	0.29	0.80	1.19	0.00

C — E232

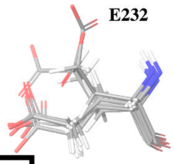

	1T4U	1T4V	3C27	4UDW	2R2M	3LDX	3SV2	1AHT	4LXB	3RM2
**1T4U**	0.00									
**1T4V**	0.39	0.00								
**3C27**	0.79	0.72	0.00							
**4UDW**	1.92	1.83	1.82	0.00						
**2R2M**	0.92	0.75	0.34	1.81	0.00					
**3LDX**	0.80	0.75	0.47	2.07	0.51	0.00				
**3SV2**	2.82	2.64	2.60	1.97	2.55	2.84	0.00			
**1AHT**	2.41	2.35	2.25	1.62	2.26	2.39	1.69	0.00		
**4LXB**	0.74	0.62	0.57	1.87	0.66	0.68	2.48	2.24	0.00	
**3RM2**	0.74	0.47	0.71	1.86	0.60	0.66	2.54	2.31	0.72	0.00

RMSD < 1	1 < RMSD < 2	2 < RMSD < 3	3 < RMSD < 4	4 < RMSD < 5	RMSD > 5

Figure S2. RMSD values (in Å) of different thrombin crystallographic structures (A), and of their residues W86 (B) and E232 (D). Conformations of residues are represented to the right.

A — B-RAF

	4XV9	5CSX	4E26	3C4C	5CSW	4YHT	3D4Q	2FB8	3PRF	4KSP
4XV9	0.00									
5CSX	1.30	0.00								
4E26	1.54	1.61	0.00							
3C4C	1.22	1.43	1.68	0.00						
5CSW	1.50	1.60	1.55	0.87	0.00					
4YHT	1.27	1.77	0.80	1.55	1.53	0.00				
3D4Q	1.50	1.89	0.86	1.85	1.81	0.56	0.00			
2FB8	1.41	1.72	0.71	1.67	1.68	0.53	0.68	0.00		
3PRF	1.48	1.85	0.80	1.64	1.73	0.59	0.54	0.61	0.00	
4KSP	1.51	1.10	1.06	1.74	1.81	1.53	1.25	1.07	1.37	0.00

B — I463

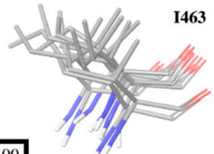

	4XV9	5CSX	4E26	3C4C	5CSW	4YHT	3D4Q	2FB8	3PRF	4KSP
4XV9	0.00									
5CSX	1.39	0.00								
4E26	1.90	2.24	0.00							
3C4C	0.83	1.69	1.66	0.00						
5CSW	1.62	1.43	1.49	1.44	0.00					
4YHT	1.62	2.00	1.12	1.24	1.35	0.00				
3D4Q	2.74	3.17	2.65	2.68	2.80	2.77	0.00			
2FB8	1.56	2.07	1.05	1.01	1.46	1.02	2.55	0.00		
3PRF	2.89	3.31	2.69	2.76	2.86	2.78	0.36	2.60	0.00	
4KSP	1.54	1.94	0.65	1.41	1.41	1.39	2.60	1.07	2.69	0.00

C — K483

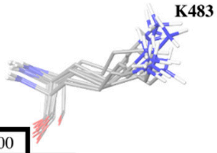

	4XV9	5CSX	4E26	3C4C	5CSW	4YHT	3D4Q	2FB8	3PRF	4KSP
4XV9	0.00									
5CSX	1.61	0.00								
4E26	2.01	1.01	0.00							
3C4C	1.38	1.26	1.49	0.00						
5CSW	1.60	1.22	1.13	1.22	0.00					
4YHT	1.60	1.40	1.34	1.08	1.70	0.00				
3D4Q	1.61	1.46	1.47	1.28	1.83	0.35	0.00			
2FB8	1.91	0.84	0.78	1.13	1.29	1.03	1.19	0.00		
3PRF	1.46	1.39	1.28	1.04	1.57	0.36	0.57	1.04	0.00	
4KSP	1.80	1.35	1.00	1.61	1.11	1.61	1.72	1.36	1.51	0.00

D — E501

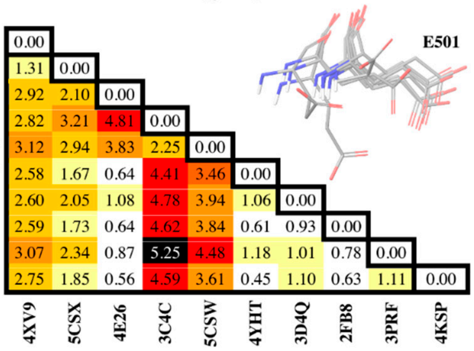

	4XV9	5CSX	4E26	3C4C	5CSW	4YHT	3D4Q	2FB8	3PRF	4KSP
4XV9	0.00									
5CSX	1.31	0.00								
4E26	2.92	2.10	0.00							
3C4C	2.82	3.21	4.81	0.00						
5CSW	3.12	2.94	3.83	2.25	0.00					
4YHT	2.58	1.67	0.64	4.41	3.46	0.00				
3D4Q	2.60	2.05	1.08	4.78	3.94	1.06	0.00			
2FB8	2.59	1.73	0.64	4.62	3.84	0.61	0.93	0.00		
3PRF	3.07	2.34	0.87	5.25	4.48	1.18	1.01	0.78	0.00	
4KSP	2.75	1.85	0.56	4.59	3.61	0.45	1.10	0.63	1.11	0.00

E — L505

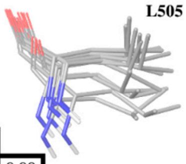

	4XV9	5CSX	4E26	3C4C	5CSW	4YHT	3D4Q	2FB8	3PRF	4KSP
4XV9	0.00									
5CSX	0.55	0.00								
4E26	1.09	1.25	0.00							
3C4C	1.17	0.84	1.94	0.00						
5CSW	1.29	0.86	1.81	0.76	0.00					
4YHT	1.00	1.33	0.49	1.99	1.98	0.00				
3D4Q	1.26	1.56	0.81	2.20	2.29	0.61	0.00			
2FB8	0.80	1.11	0.60	1.82	1.72	0.48	0.98	0.00		
3PRF	1.39	1.68	0.75	2.35	2.39	0.61	0.37	0.94	0.00	
4KSP	1.18	1.34	0.41	2.00	1.98	0.60	0.54	0.83	0.52	0.00

F — F583

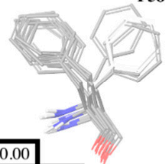

	4XV9	5CSX	4E26	3C4C	5CSW	4YHT	3D4Q	2FB8	3PRF	4KSP
4XV9	0.00									
5CSX	2.53	0.00								
4E26	3.31	3.94	0.00							
3C4C	3.63	4.22	2.27	0.00						
5CSW	3.07	3.83	0.58	2.34	0.00					
4YHT	3.26	3.90	2.25	0.71	2.26	0.00				
3D4Q	3.19	4.04	2.31	1.12	2.32	0.66	0.00			
2FB8	3.28	4.05	2.33	0.73	2.32	0.46	0.54	0.00		
3PRF	3.43	4.19	2.34	0.73	2.38	0.57	0.46	0.34	0.00	
4KSP	1.10	2.22	3.70	4.20	3.50	3.81	3.79	3.92	4.04	0.00

RMSD < 1	1 < RMSD < 2	2 < RMSD < 3	3 < RMSD < 4	4 < RMSD < 5	RMSD > 5

Figure S3. RMSD values (in Å) of different B-RAF crystallographic structures (A), and of their residues I463 (B), K483 (C), E501 (D), L505 (E) and F583 (F). Conformations of residues are represented to the right.
